# Influenza A virus diffusion through mucus gel networks

**DOI:** 10.1038/s42003-022-03204-3

**Published:** 2022-03-22

**Authors:** Logan Kaler, Ethan Iverson, Shahed Bader, Daniel Song, Margaret A. Scull, Gregg A. Duncan

**Affiliations:** 1 Biophysics Program, University of Maryland, College Park, MD USA; 2 Department of Cell Biology & Molecular Genetics, University of Maryland, College Park, MD USA; 3 Fischell Department of Bioengineering, University of Maryland, College Park, MD USA

**Keywords:** Nanoscale biophysics, Influenza virus

## Abstract

Mucus in the lung plays an essential role as a barrier to infection by viral pathogens such as influenza A virus (IAV). Previous work determined mucin-associated sialic acid acts as a decoy receptor for IAV hemagglutinin (HA) binding and the sialic-acid cleaving enzyme, neuraminidase (NA), facilitates virus passage through mucus. However, it has yet to be fully addressed how the physical structure of the mucus gel influences its barrier function and its ability to trap viruses via glycan mediated interactions to prevent infection. To address this, IAV and nanoparticle diffusion in human airway mucus and mucin-based hydrogels is quantified using fluorescence video microscopy. We find the mobility of IAV in mucus is significantly influenced by the mesh structure of the gel and in contrast to prior reports, these effects likely influence virus passage through mucus gels to a greater extent than HA and NA activity. In addition, an analytical approach is developed to estimate the binding affinity of IAV to the mucus meshwork, yielding dissociation constants in the mM range, indicative of weak IAV-mucus binding. Our results provide important insights on how the adhesive and physical barrier properties of mucus influence the dissemination of IAV within the lung microenvironment.

## Abstract (web summary)

Influenza A virus movement in mucus is found to be affected by the mesh structure of the gel network and further analysis reveals weak IAV-mucus binding.

## Introduction

Influenza A virus (IAV) is a constantly evolving pathogen associated with significant morbidity and mortality, making it a persistent threat to public health^1,2^. Individuals with chronic lung disease, including asthma and chronic obstructive pulmonary disease, have an increased risk of potentially life-threating medical complications due to influenza virus infection^3,4^. To protect the respiratory tract from infection by the influenza virus and other pathogens, a thin layer of mucus is constantly produced and coats the surface of the airway epithelium^5^. Mucus is a hydrogel composed of approximately 98% water and 2% solids, containing secreted mucins, globular proteins, lipid surfactants and cellular debris^6,7^. The primary building blocks of mucus gels are high molecular weight (≥1 MDa) mucin biopolymers that are heavily glycosylated with terminal groups such as fucose, sulfate, and sialic acid^7–9^. Mucin biopolymers interweave to form a gel network with a pore size ranging from 100 to 500 nm, depending on total mucin concentration^8^. Ciliated cells on the airway epithelium beat in a coordinated fashion to clear mucus and trapped particulates from the airway^10^. In order to prevent infection, mucus in the lung must effectively trap inhaled pathogens and the mechanisms by which this is achieved are important to our understanding of innate host defenses.

The IAV lipid envelope contains receptor-engaging hemagglutinin (HA) glycoproteins and receptor-destroying neuraminidase (NA) enzymes^11^. HA binds to α-2,3 and/or α-2,6 linked sialic acid on the surface of the cell to allow for internalization of the virus^12,13^. The various strains of influenza virus have different sialic acid-binding preference, with human viruses typically preferring α-2,6 linked sialic acid while avian and equine viruses prefer α-2,3 linked sialic acid^14^. Newly formed virions are released from the cell when NA catalyzes the cleavage of terminal sialic acid^15^. Previous work has shown the same HA/NA machinery also plays a role in the penetration of IAV through the mucus barrier^13,16,17^. Specifically, prior work has shown HA on IAV binds to sialic acid within mucus, which is predominantly α-2,3 linked sialic acid on mucins^18^. It has been proposed that constant NA activity is required for viral penetration through mucus to facilitate removal of sialic acid decoy receptors^17^. However, whether this can be generalized to human influenza virus infections remains unclear as the evidence to support this mechanism is largely based on data generated using nonhuman IAV, non-human mucus, and synthetic glycan-coated substrates. In addition, prior work on nanoparticle and virus diffusion has noted substantial variation between the barrier properties of human mucus between individual donors^19–21^, which would likely contribute to differences in their susceptibility to infection. It has yet to be established if host-specific mucus barrier properties, such as mucus gel pore size and/or mucin glycan profile, influence the trapping and elimination of IAV from the respiratory tract.

In this work, we use fluorescence microscopy to measure the diffusion of IAV through human mucus. Our investigations were conducted using the well-characterized H1N1 influenza A/Puerto Rico/8/34 (PR8) strain, of human origin but with an ‘avian-like’ HA binding preference for α-2,3 linked sialic acid^22^. PR8 IAV was chosen for these studies given its widespread use and reported binding preference for α-2,3 sialic acid which we anticipate are present in abundant quantity in human mucus^18^. We used mucus collected from endotracheal tubes of 10 donors for our studies to assess variation in their barrier properties. An important component of our work was also measuring diffusion of nanoparticles designed to be minimally adhesive to mucus^19,23^ with a comparable size to IAV (~120 nm) in each sample tested. This enabled us to develop an analytical model to decouple physical and adhesive barrier functions of mucus towards IAV. This technique provides a method to assess effective dissociation constants for IAV and potentially other respiratory viruses in 3D human mucus gels. The results of this work give insight into how the structural and adhesive properties of mucus influence its protective function against IAV infection.

## Results

### IAV diffusivity in human mucus

**Fig. 1:**
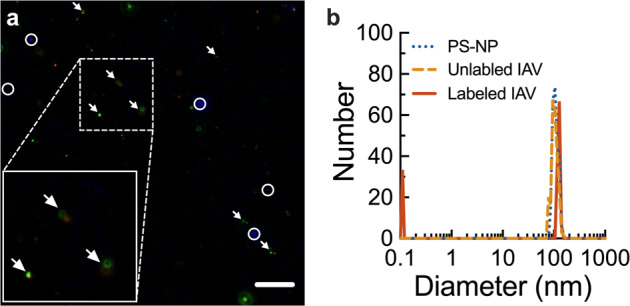
Characterization of fluorescent IAV. **a** Fluorescence micrograph of purified IAV and 100 nm PS-NP (blue) stained with DiI (orange) and anti-HA antibody (green). DiI and anti-HA co-staining is denoted with white arrows. PS-NP indicated by white circles. Scale bar = 10 µm. **b** Measured hydrodynamic diameter for muco-inert PS-NP (dotted blue), unlabeled IAV(dashed orange), and DiI-labeled IAV (solid red).

To visualize IAV in human mucus, we used the lipophilic dye, 1,1′-dioctadecyl-3,3,3′3′-tetramethylindocarbocyanine perchlorate (DiI), to label individual virions. To confirm the identity of fluorescently tagged particles, DiI-labeled IAV were stained with an antibody specific to IAV HA and imaged using fluorescence microscopy, revealing co-localization of the dye and viral antigen (Fig. 1a). Nanoparticles were included in the imaging to establish plane of view. We next used dynamic light scattering (DLS) to compare the size of unlabeled and DiI-labeled IAV. Our results indicate both labeled and unlabeled IAV were ~120 nm in diameter, suggesting that DiI intercalation did not disrupt virions or cause particle aggregation (Fig. 1b). Further, we confirmed DiI labeling did not impact IAV infectivity (Supplementary Fig. 1).

**Fig. 2:**
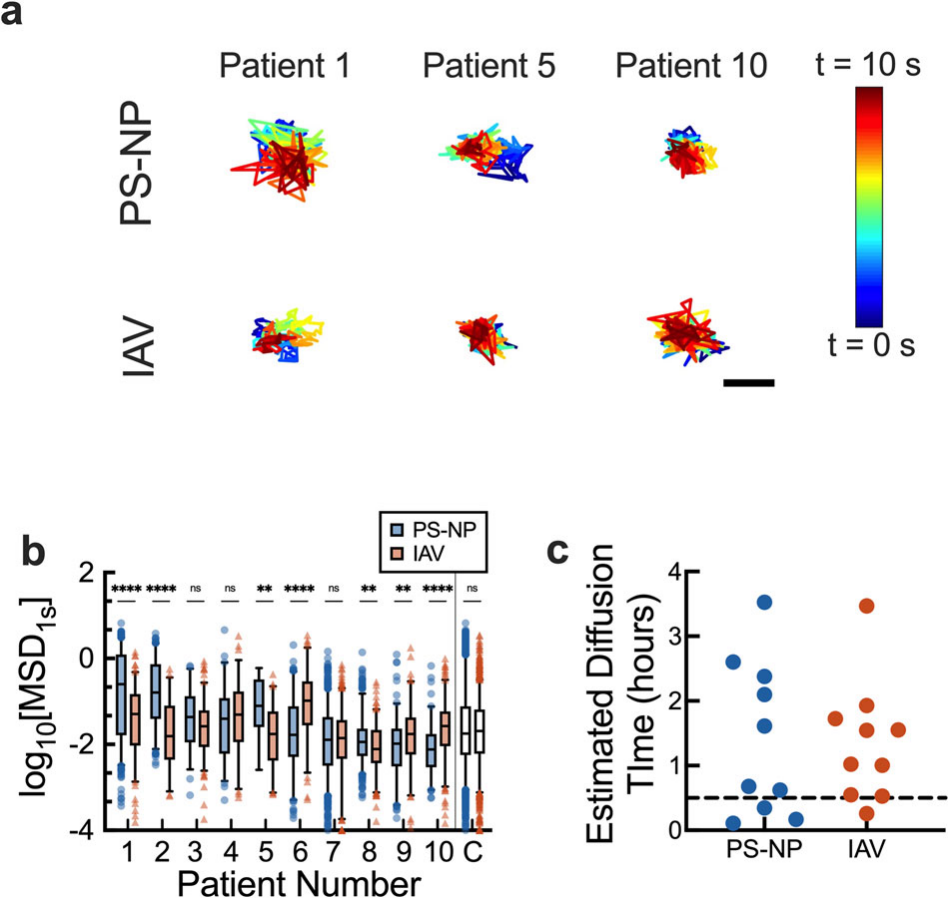
Diffusion of IAV and nanoparticles with similar diameter in human mucus. **a** Representative trajectories of polyethylene glycol (PEG) coated 100 nm polystyrene nanoparticles (PS-NP) and IAV diffusion in mucus. Traces show 10 seconds of motion with a color scale to indicate time. The scale bar represents 0.2 µm. **b** Box-and-whisker plots of log-based 10 of MSD at τ = 1 s (log_10_MSD) PS-NP (blue circles) and IAV (red triangles) in mucus samples collected from 10 individual patients are shown. The combined data set for all samples tested (C) is also shown. Patients are numbered in descending order according to the median MSD of PS-NP particles in each sample. **c** Estimated diffusion time calculated from the average effective diffusivity (*D*) for PS-NP (blue) and IAV (red) particles to diffuse through a 7 µm thick mucus layer. Dotted line at 30 min (0.5 hours) indicates cutoff to avoid removal due mucociliary clearance. Whiskers are drawn down to the 5th percentile, up to the 95th percentile, and the outliers are shown as points. Data sets (*n* = 10 patient samples) statistically analyzed with two-tailed Mann-Whitney test: ns = not significant; *p* > 0.05, *p < 0.05, **p < 0.01, ***p < 0.001, ****p < 0.0001.

After confirming successful IAV labeling, we introduced DiI-labeled IAV and muco-inert polystyrene nanoparticles (PS-NP) to human airway mucus and measured their diffusion using fluorescence video microscopy and multiple particle tracking (MPT) analysis. By simultaneously measuring PS-NP and IAV diffusion, we were able to analyze mucus microrheology and determine the diffusion rate of IAV particles within the same regions of interest. The diffusivity, as measured by logarithm based 10 of mean squared displacement (MSD) at a time lag of 1 s (log_10_[MSD_1s_]), showed significant differences between PS-NP and IAV diffusion for individual samples (Fig. 2a, b). Measured MSD for IAV varied over several orders of magnitude between individual patient samples. However, when comparing across all samples collected as shown in the combined data set (Fig. 2b; column ‘C’), we found IAV and PS-NP diffusion are comparable. To put these results into context, we calculated the average effective diffusivity (D) for each sample which was then used to estimate the time necessary for the particles to diffuse (considering motion in z-direction only) through an airway mucus layer with a physiological thickness of 7 µm (Fig. 2c). We would expect IAV capable of penetrating the mucus gel in under 30 min, where *D* > 0.01 µm/s, would reach the airway epithelial surface and avoid removal via mucociliary clearance^10^. Based on these estimates, we find that IAV would penetrate the mucus barrier in ≤30 min for 3 out of the 10 samples tested and as a result, would be more likely to cause infection.

### Measuring an effective dissociation constant for IAV binding to human mucus gels

**Fig. 3:**
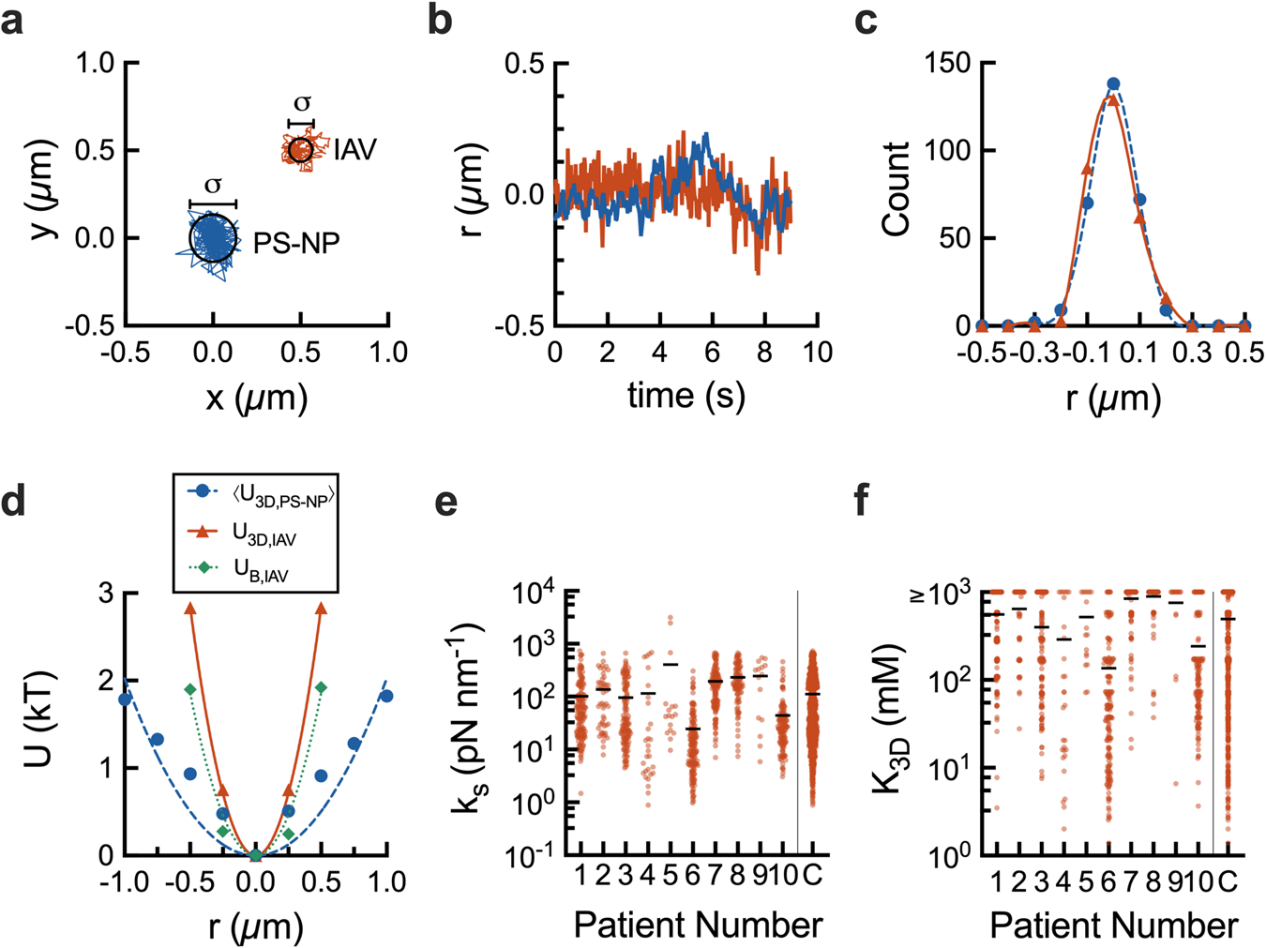
Quantifying IAV binding to human mucus. **a** Representative PS-NP (blue) and IAV (red) trajectories centered around the average x and y value. IAV trajectory is offset by 0.5 µm in the x and y-direction. Average diameter of trajectories is indicated by black circles and labeled *σ*. **b** Radial position from the trajectory center (*r*) versus time for a single PS-NP (blue) and IAV (red) particle. **c** Histogram of sampled *r* for a single PS-NP (blue circles) and IAV (red triangles) particle. **d** Energy (*U*) versus radial position (*r*) for the average PS-NP energy of confinement for one video (〈*U*_3D,NP_〉, blue circles), an individual IAV particle’s energy of confinement (*U*_3D,IAV_, red triangles), and energy of IAV-mucus binding for the same IAV particle (*U*_B,IAV_, green diamonds). Scatter plots of calculated (**e**) spring constant (*k*_s_), and **f** dissociation constant (*K*_3D_) for individual IAV particles. In **e** and **f**, combined data for all samples is denoted as (C). Line drawn at mean value. For **f**, IAV with a calculated *K*_3D_ ≥ 1000 mM are compiled with these high *K*_3D_ indicative of negligible IAV-mucus interactions. Patient numbers correspond with those in Fig. 2.

To directly measure IAV interactions with glycans within the mucus gel network, we further analyzed IAV trajectories using statistical mechanics. The step-by-step procedure, representative potentials, and resulting binding affinities for IAV are shown in Fig. 3. IAV and PS-NP trajectories (Fig. 3a) are transformed to radial positions with respect to the trajectory center (Fig. 3b, c). For IAV, we define an equilibrium energy of confinement (*U*_3D,IAV_) as the sum of interactions resulting from gel network viscoelasticity and receptor-mediated glycan binding. Given the comparable size of IAV and PS-NP (~120 nm), we used PS-NP as micromechanical ‘gauges’ of network viscoelasticity as experienced by IAV. Importantly, PS-NP have been designed to exhibit negligible interactions with mucins^24^. In order to decouple the influence of physical confinement (*U*_s_) and mucin glycan binding on IAV diffusion (*U*_B,IAV_), we subtract the average measured *U* for PS-NP 〈*U*_3D,NP_〉 from measured *U* for IAV (*U*_3D,IAV_) to effectively remove the influence of local gel mechanics, as shown in Fig. 3d. With this accounted for, we fit *U*_B,IAV_ energy profiles with a harmonic well potential representation and a general expression was used to estimate a dissociation constant for individual IAV^25^. Using this approach, the IAV trajectories were analyzed to determine the spring constant (*k*_s_) and effective dissociation constant (*K*_3D_) for binding of IAV to human mucus (Fig. 3e, f). Based on our analysis, we find the mode and median for *k*_s_ of 38 and 43 pN nm^−1^, and *K*_3D_ of 163 and 300 mM, respectively.

### Effect of IAV neuraminidase (NA) inhibition on IAV diffusion

**Fig. 4:**
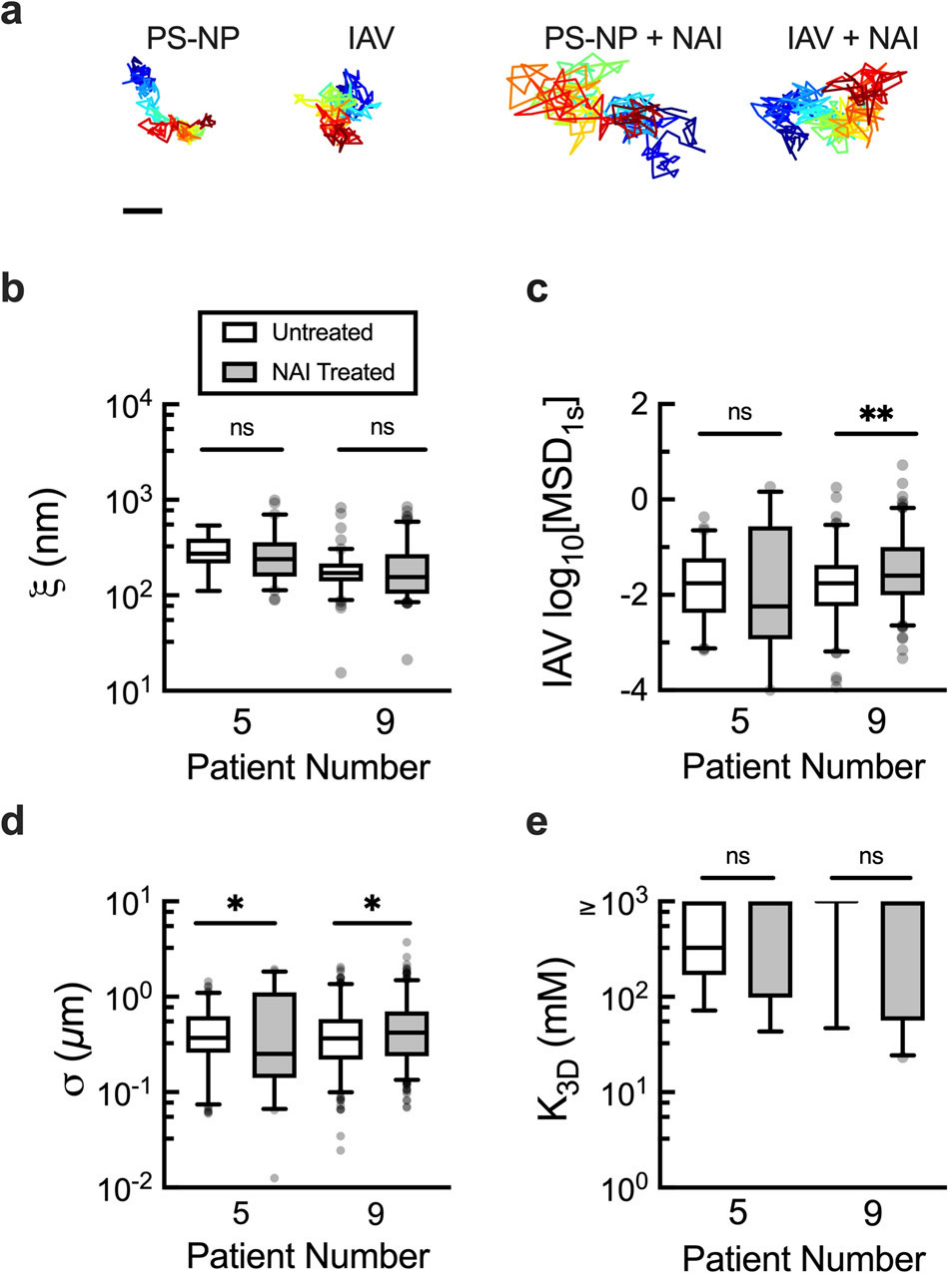
The effect of neuraminidase (NA) inhibition on IAV diffusion through and adhesion to human mucus. **a** Representative trajectories of PS-NP and IAV untreated and treated with NAI in human mucus. Scale bar = 0.2 µm. **b** Calculated pore size (*ξ*) in untreated (white) and NA inhibitor (NAI) treated (zanamivir; 10 µM final concentration; grey) based on PS-NP diffusion in human mucus. **c** Measured log_10_MSD_1s_ for IAV diffusion in untreated and NAI treated human mucus. Box and whisker plots of **d** average trajectory diameter (*σ*) and **e** calculated dissociation constants (*K*_3D_) with and without NAI treatment. IAV with a calculated *K*_3D_ ≥ 1000 mM are compiled with these high *K*_3D_ indicative of negligible IAV-mucus interactions. Whiskers are drawn down to the 5th percentile, up to the 95th percentile, and outliers are plotted as points. Data sets (*n* = 2 patient samples) statistically analyzed with two-tailed Mann-Whitney test: ns = not significant; *p* > 0.05, *p < 0.05, **p < 0.01. Patient numbers correspond with those in Figs. 2 and 3.

Given these measurable effects of glycan binding on IAV diffusion, we next examined the impact of NA activity on IAV diffusion. Prior work has shown IAV is immobilized when NA activity is inhibited given it will not be able to cleave bonds between IAV HA and mucin-associated sialic acid^13,18^. To test this, we measured IAV diffusion in human mucus in the presence of the NA inhibitor (NAI), zanamivir (Fig. 4). Control experiments confirmed NA activity is completely abolished by NAI at a final concentration of 10 µM (Supplementary Fig. 2) and the addition of NAI to IAV did not result in viral aggregation (Supplementary Fig. 3). Thus, samples containing IAV and PS-NP were treated with 10 µM NAI, imaged, and subsequently analyzed with MPT. When comparing samples from 2 donors that exhibited no significant change in pore size after NAI treatment, we observed the diffusivity of the IAV was unchanged in 1 sample (patient 5) and significantly increased in the other sample (patient 9) in the context of NA inhibition (Fig. 4a-c). We also found a significant change in the average trajectory diameter (*σ*) for IAV in both samples while the *K*_3D_ values showed no significant change (Fig. 4d, e). Unexpectedly, two additional samples treated with NAI showed a significant change in gel pore size which presents a challenge in interpreting the effects of NA inactivation. The data for these samples are provided in the supplemental information (Supplementary Fig. 4).

### Disruption of mucus gel cross-linking and its impact on IAV diffusion

**Fig. 5:**
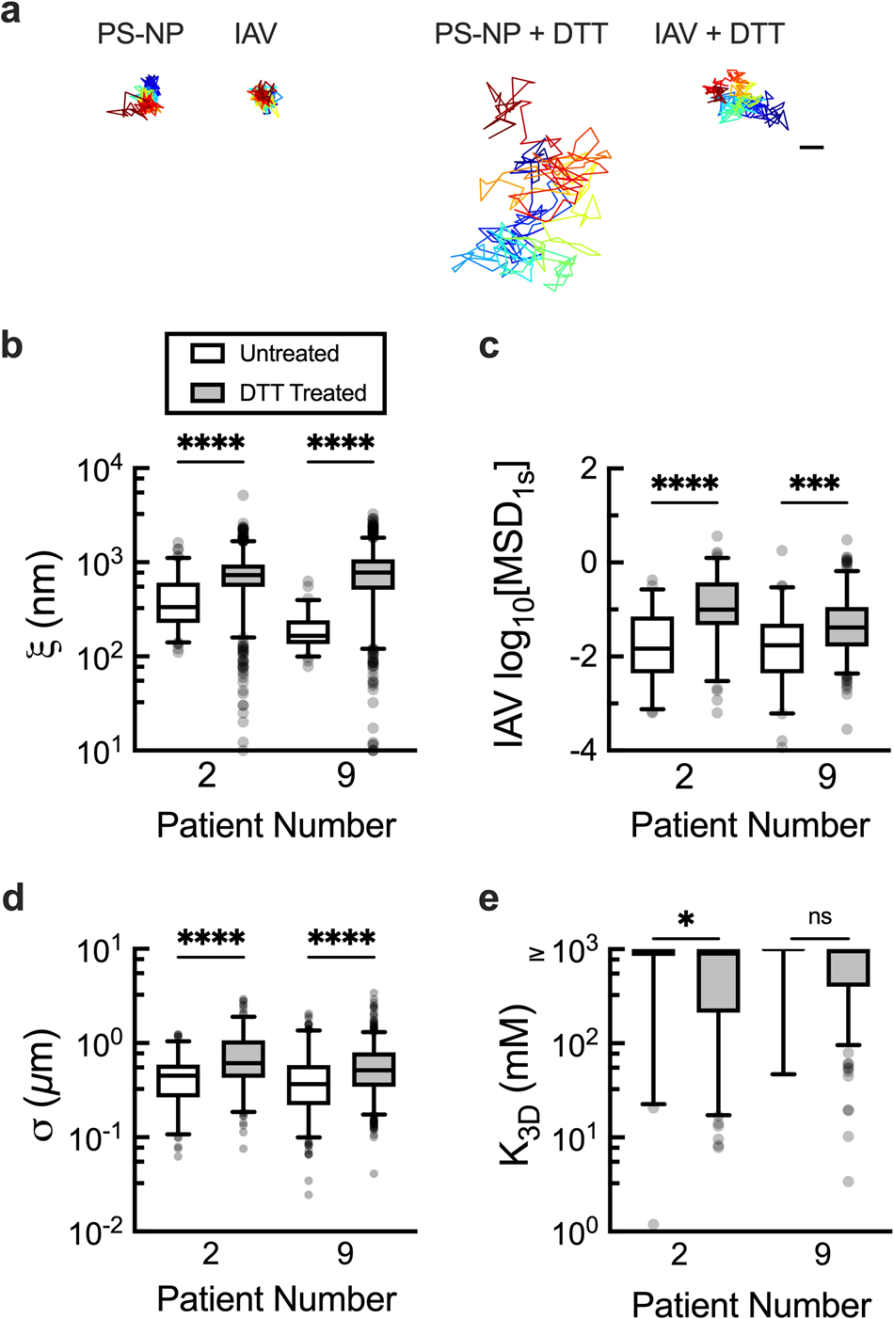
Impact of reducing disulfide bonds within mucus gels on IAV diffusion and adhesion. **a** Representative trajectories of PS-NP and IAV diffusion in mucus with and without DTT treatment. Scale bar = 0.2 µm. **b** Calculated mucus gel pore size (ξ) based on PS-NP diffusion in untreated (white) and DTT treated (grey) mucus (5 mM final concentration). **c** Measured log_10_MSD_1s_ for IAV in untreated and DTT-treated mucus. Box and whisker plots of **d** average trajectory diameter (*σ*), and **e** calculated dissociation constant (*K*_3D_) for individual IAV particles with and without DTT treatment. For **e**, IAV with a calculated *K*_3D_ ≥ 1000 mM are compiled with these high *K*_3D_ indicative of negligible IAV-mucus interactions. Whiskers are drawn down to the 5th percentile, up to the 95th percentile, and outliers are plotted as points. Data sets (*n* = 2 patient samples) statistically analyzed with two-tailed Mann-Whitney test: ns = not significant; *p* > 0.05, *p < 0.05, **p < 0.01, ***p < 0.001, ****p < 0.0001. Patient numbers correspond with those in Figs. 2–4.

We next investigated the effect of cross-linking on IAV diffusion by treating samples with the reducing agent, dithiothreitol (DTT). We reasoned that reducing disulfide cross-linking in human mucus would result in larger network pores and allow for IAV to more readily diffuse through human mucus. Importantly, control experiments confirmed that treatment with 5 mM DTT does not impact viral infectivity (Supplementary Fig. 5). To examine IAV diffusion after disulfide bond disruption, samples containing IAV and PS-NP were treated with DTT and analyzed using MPT. Once treated, both PS-NP and IAV traveled significantly greater distances (Fig. 5a) leading to the significant increase in measured pore size (Fig. 5b), and diffusion rate of the IAV particles (Fig. 5c). Additionally, we analyzed the trajectories of the IAV to determine *σ* and *K*_3D_ for the DTT treated samples compared to the untreated samples. In doing so, we observed a significant increase in *σ* (Fig. 5d) and decrease in *K*_3D_ (Fig. 5e) after DTT treatment and disulfide bond reduction.

### Diffusion of IAV in synthetic mucus with altered microstructural properties

**Fig. 6:**
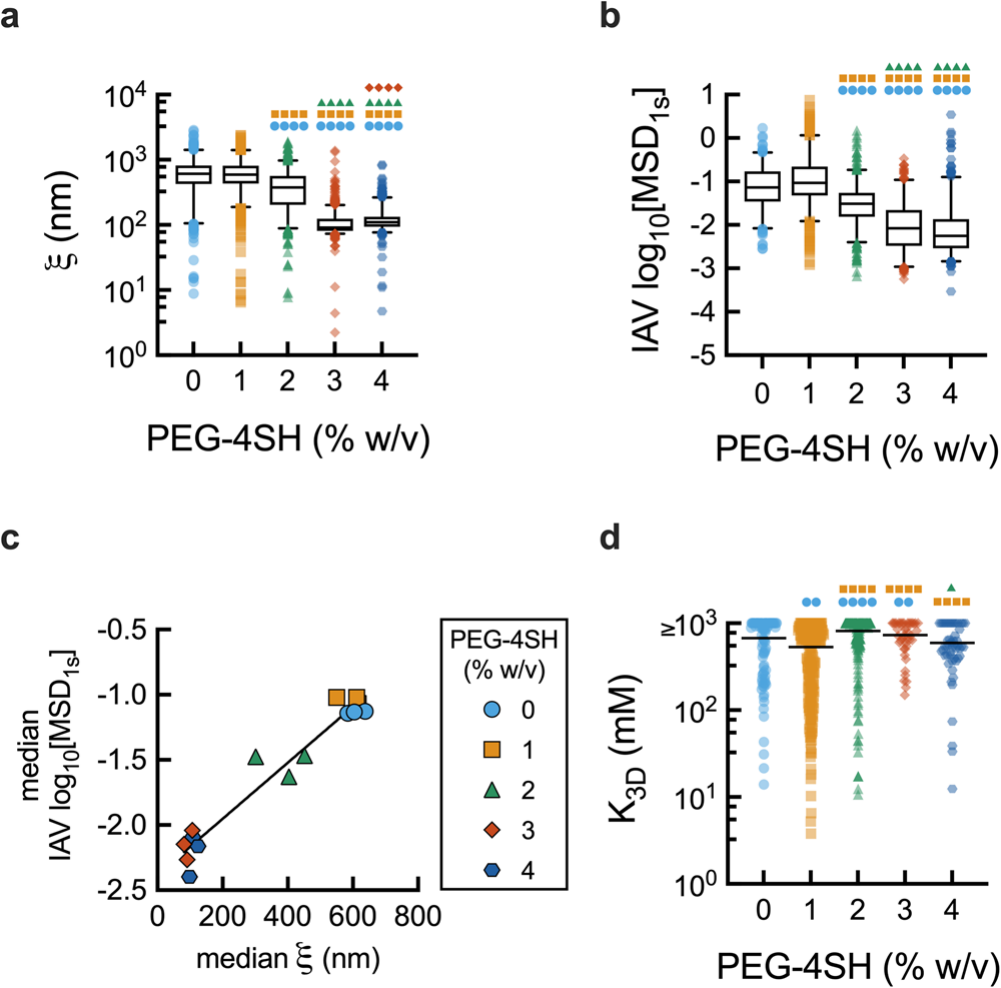
Modulation of crosslinking density effects IAV diffusion through synthetic mucus. **a** Calculated mucus gel pore size (ξ) based on PS-NP diffusion in synthetic mucus. **b** Measured log_10_MSD_1s_ for IAV in synthetic mucus. **c** Median gel pore size (ξ) calculated from the PS-NP compared to the median log_10_ MSD value for the IAV particles in synthetic mucus of varying crosslinking densities, *R*^2^ = 0.9435. **d** Calculated dissociation constant (*K*_3D_) for individual IAV particles, the line at mean value for each data set. IAV with a calculated *K*_3D_ ≥ 1000 mM are compiled with these high *K*_3D_ indicative of negligible IAV-mucus interactions. Whiskers are drawn down to the 5th percentile, up to the 95th percentile, and outliers are plotted as points. Data sets (*n* = 3 synthetic hydrogels per group) statistically analyzed with Kruskal-Wallis test and Dunn’s test for multiple comparisons: *p < 0.05, **p < 0.01, ***p < 0.001, ****p < 0.0001. Color and symbol indicative of comparison group: 0%, light blue circles; 1%, orange squares; 2%, green triangles; 3%, red diamonds; 4%, dark blue hexagons.

We then investigated the relationship between mucus crosslinking and IAV diffusion using a synthetic mucus model^26^. We hypothesized that a systematic increase in synthetic mucus gel crosslinking and resulting tightening of gel pore structure would lead to a reduction in IAV diffusion rates. To test this, MPT experiments were performed on IAV and PS-NP in synthetic mucus of varying crosslinking densities. The total mucin concentration and associated sialic acid content were kept constant in all cases. As the crosslinker concentration increased, the network pore size within the synthetic mucus decreased (Fig. 6a), which was mirrored in the decreased diffusivity of the IAV (Fig. 6b). Comparison of measured pore size based on microrheology using PS-NP and the log_10_MSD values for the IAV revealed that increased pore size correlated with increased diffusivity (Fig. 6c). Additionally, while there are significant differences in the calculated dissociation constant (*K*_3D_) for the IAV, we observed a consistent range of mean *K*_3D_ values across all crosslinking percentages (Fig. 6d).

## Discussion

In this work, we examined the additive and synergistic roles of gel network structure and IAV-glycan binding in the trapping of viral particles in human mucus. Consistent with prior studies^13,27^, IAV displayed lower mobility than muco-inert PS-NP with the comparable diameter (~120 nm) in 5 out of the 10 samples, presumably as a result of IAV binding to the mucus gel network. However in the other 5 samples tested, we observed IAV more rapidly diffused through mucus than PS-NP. Previous work has demonstrated rigidity-dependent diffusion of lipid and protein-based nanoparticles through biological gels where particle deformability can be tuned to enhance mobility^28,29^. As this seemed to occur in more densely cross-linked mucus gels with pore sizes below 200 nm (Supplementary Fig. 6), this may be a result of the semi-deformable nature of IAV particles^30^ compared to rigid PS-NP, but additional work is required to explore this. However overall, diffusion of IAV and PS-NP was comparable when considering all samples tested. To further characterize IAV-mucus interactions, we developed a statistical mechanics-based analysis to decouple the impact of network structure and glycan binding on IAV diffusion behavior in human mucus. The effective dissociation constant (*K*_3D_) determined for individual IAV virions in all human mucus samples tested were within a mM range, consistent for protein-sugar mediated binding^31,32^. These relatively weak IAV-mucus interactions alone are unlikely to provide sufficient means to immobilize IAV, but likely contribute to the slowing of IAV diffusion as compared to the non-mucoadhesive PS-NP as seen in half of the donor samples in Fig. 2b. We also found disrupting mucus crosslinking with the reducing agent DTT enhanced IAV diffusion through mucus due to the increased network pore size. Interestingly, disruption of the mucus gel via DTT treatment also resulted in a broader range and effective reduction in *K*_3D_ values, indicative of greater IAV-mucus association. Further, we have demonstrated that a smaller network size, as detected by PS-NP, substantially decreases IAV mobility in a synthetic mucus model with systematically varied crosslinking density.

We have also developed an analysis capable of extracting effective binding constants for IAV within the mucus gel network. Unlike standard approaches using glycan binding arrays with purified viral and glycan components^33,34^, we are able to directly determine effective disassociation constants (*K*_3D_) for intact IAV within the 3D microenvironment of native and synthetic mucus gels. Importantly, our analysis allows us to account for the physical pore structure of mucus gels with analysis of PS-NP confinement energies. The calculated *k*_s_ for IAV-mucus binding, with the median value of 43 pN/nm, is on the order of forces required to disrupt HA-sialic acid bonds (10-25 pN^35,36^). Calculated *K*_3D_ values in human mucus varied widely between patients over a mM range, but are generally consistent with short-lived, reversible HA-sialic acid bonds. In previous work, the dissociation constants for recombinant X-31 HA protein from A/Aichi/2/1968 (H3N2) were quantified using nuclear magnetic resonance imaging. In the presence of α2,3-linked sialyllactose (3′) sialyllactose and α2,6-linked (6′) sialyllactose, the *K*_D_ values for the HA protein were 3.2 mM and 2.1 mM, respectively^37^. We attribute the large range in measured *K*_3D_ to differences in the properties of mucus collected rather than functional differences in individual IAV. There is likely wide variation in the glycans present in human mucus from different donors.

These estimates of *K*_3D_ would suggest IAV HA only weakly associates with the mucus network. A potential explanation for this finding is the nature of sialic acid linkages in mucus gels as compared to the surface of the airway epithelium. More specifically, mucin-associated sialic acid are generally O-linked whereas they are present predominantly in an N-linked form on the epithelial surface which may influence IAV recognition and binding^38^. In addition, free sialic acid and other sialylated soluble factors (e.g., H-ficolin^39^) present within mucus could competitively inhibit IAV binding to sialic acid groups anchored to the mucus gel. To further examine the impact of IAV binding to sialic acid on mucus penetration, we performed studies on the diffusion of IAV in mucus after pre-treatment with 3’ and 6’ sialyllactose to competitively inhibit these interactions with the mucus mesh (Supplementary Fig. 7). However, based on our analyses, sialyllactose treatment of IAV did not have a substantial impact on IAV diffusion and association with the mucus network.

Interestingly, while we found that treatment with NAI resulted in a significant increase in the average trajectory diameter (*σ*) for 1 of the samples, there was no significant change in *K*_3D_ values. This would indicate that NA has a small influence on the binding affinity of IAV and HA binding alone is unlikely to lead to irreversible bond formation. While our results indicate the low binding affinity of HA, we should also note HA/NA cooperativity may play a role in the association of IAV to the mucus gel^40^. We also considered the possibility that IAV NA could degrade mucus and disrupt its structure to promote penetration, similar to the mechanism used by bacterial pathogens^41^. However, we observed an increase in PS-NP diffusion when incubated with IAV in only 1 of 3 patient samples tested (Supplementary Fig. 8). We also found that while we observed a significant increase in *σ*, the range of *K*_3D_ values decreased following disruption of disulfide bonds which we attribute to the increased availability of binding sites on mucin polymers to engage the following disruption of the gel network. This may be explained, at least in part, by sterically limited HA-sialic acid binding within dense and/or highly crosslinked mucus given the estimates of *K*_3D_ ≥ 1000 mM, indicative of negligible HA-mediated glycan binding. This is further substantiated by the slight reduction in *K*_3D_ values observed in the synthetic mucus gels with lower percentages of crosslinker (<2% w/v PEG-4SH). Relevant to the above-mentioned observations, a recent study demonstrated the inhibitory effect of high mucin density on IAV binding to supported lipid bilayers engineered with surface-tethered synthetic mucin mimetics^42^. In this previous work, IAV attachment to sialylated mucin mimics was apparent but a reduction in viral attachment was observed above a critical concentration suggesting steric limitation of IAV binding. Using a 3D synthetic mucus gel network, we also observed a slight increase in *K*_3D_ values (i.e., less IAV-mucus binding) as the percentage of crosslinker increased.

Several reports on IAV diffusion through mucus suggest interactions with mucin-associated glycans contribute to its transport behavior^13,18,43^. For example, it has been shown H1N1 and H3N2 IAV adhere to human lung tissue sections and salivary mucus in a sialic acid-dependent manner^16^. In addition, previous work has shown NA activity of a swine IAV facilitates its transit through porcine lung mucus^13^. Conversely, we have found in our studies NA inactivation does not reduce IAV diffusion through mucus. The unexpected increase in network size and IAV mobility observed after NAI treatment (Supplementary Fig. 4) may be explained by either direct effects of NAI on mucus structure or differences between aliquots from individual donors. Based on our results, it is likely IAV HA binding of mucin glycans is weak and reversible in the presence or absence of NA activity. In a previous study, Wang et al. also found that IAV penetration through mucus was not dependent on NA activity, but rather IAV was immobilized in mucus by influenza-specific IgG antibodies^27^. We note this work was performed similarly to our work with the use of the PR8 strain of IAV in human mucus collected from endotracheal tubes using multiple particle tracking to quantify IAV diffusion rate. However, we did not find evidence of uniform IgG-mediated trapping in our work presumably as the mucus samples collected from individuals during our study from 2018 to 2020 are unlikely to contain antibodies strongly reactive to the lab-adapted PR8 strain initially isolated in 1934. More recent work by Vahey & Fletcher showed that NA-enhanced influenza A transport was limited to nonspherical, filamentous viral particles as opposed to their spherical counterparts^44^. Given PR8 forms predominantly spherical virions, our results on the limited impact of NA align with these previous studies.

The literature to date has generally focused on glycan-mediated entrapment of IAV within human mucus^9,13,18^. From a more physiological perspective, our data support the rationale that the crosslinking and pore structure of mucus gels can help to protect the airway epithelium from IAV and potentially other respiratory viruses. The inflammatory response to IAV infection is known to increase reactive oxygen species (ROS) production and cytokine expression resulting in acute lung injury^45^. In response to ROS and oxidative stress, the epidermal growth factor (EGFR) pathway, responsible for mucin hypersecretion, becomes activated leading to increased mucus concentration in the airway lumen^46,47^. In addition, prior studies have found ROS and oxidative stress can increase the elasticity of airway mucus, attributed to the oxidation of mucin cysteine domains leading to the formation of mucin-mucin disulfide crosslinks^48^. Increased mucin concentration and mucin-mucin crosslinking due to oxidative stress might be a beneficial part of the inflammatory response towards IAV infection to hinder virus penetration through the mucus barrier. These potential benefits, however, may be undermined by compromised airway clearance in mucus gels with abnormal viscous and elastic properties^10^.

While we think our studies using patient-derived lung mucus provides a physiologically relevant system to investigate IAV dynamics in the airway microenvironment, we acknowledge the modest sample size limited by their availability and note that all experiments were performed with only the PR8 strain. To determine the broader relevance of these findings, we plan to include alternative strains in future work with different sialic acid preference and more contemporary IAV strains where neutralizing antibody-mediated entrapment may influence IAV-mucus interactions as has been observed in prior work^27^. We also note PR8 and other lab adapted IAV strains produce primarily spherical virions^49^. In natural infections, influenza virions can vary in morphology, producing a mixture of spherical and filamentous virions^49^. Recent work has also shown the impact of IAV particle morphology on transport in mucus which will be important to consider in subsequent studies^44^. Based on this prior work, IAV with a filamentous or anisotropic shape with the potential for HA/NA polarization within the IAV envelope are more likely to engage in multivalent HA-sialic acid interactions than spherical IAV like the PR8 strain used in this work.

In summary, we have determined that IAV mobility can be limited by both the structural and biochemical features of the mucus gel network. The influence of gel network structure observed in our work bears similarity to past studies that demonstrate the size dependence of particle diffusion through mucus and other biological matrices^28,50–53^. Unlike previous reports, we found HA on the IAV envelope exhibited negligible binding to sialic acid, indicating that PR8 IAV penetration through dense mucus is not dependent solely on NA function. As might be expected, mucus gels with pore sizes approaching the diameter of IAV are capable of physically entrapping viral particles. Our measurements suggest IAV binding to mucus is weak and reversible due to low-affinity interactions between HA and sialic acid, which likely improves its ability to navigate to the underlying airway epithelium. Steric hindrance within the mucus gel network may also influence the accessibility of sialic acid receptors to HA binding. Our results also provide a framework for direct measurement of viral particle association to mucus gels which may prove useful for future studies on respiratory viruses by our group and others in the field.

## Methods

### IAV and nanoparticle preparation

The plasmid-based reverse genetics system for influenza viruses A/Puerto Rico/8/34 (PR8; H1N1) was a gift from Adolfo Garcia-Sastre. Infectious virus was generated from cloned cDNAs in 293 T and Madin-Darby canine kidney (MDCK) cell co-cultures and purified by centrifugation through a 20% sucrose cushion as previously described^54,55^. The infectivity of resulting virus stocks was quantified by standard plaque assay on MDCK cells^56^, yielding 3.9 × 10^9^ plaque-forming units (PFU)/mL for PR8. IAV was subsequently labeled with a lipophilic dye, 1,1′-dioctadecyl-3,3,3′3′-tetramethylindocarbocyanine perchlorate (DiI; Invitrogen). The labeled virions were then purified and concentrated via haemadsorption to chicken red blood cells^57^. Final DiI-labeled IAV stocks were aliquoted and stored at −80 °C. Each aliquot underwent a maximum of two freeze thaws. This strain exhibits a primarily spherical morphology and diameter of roughly 120 nm. DiI-labeled virus stocks were counter-stained with polyclonal anti-influenza virus H1 (H0) HA PR8 antibody (NR-3148; antiserum, goat; BEI Resources, NIAID, NIH). Carboxylate modified fluorescent PS nanoparticles (PS-NP; Life Technologies) with a diameter of 100 nm were coated with a high surface density of polyethylene glycol (PEG) via a carboxyl-amine linkage using 5-kDa methoxy PEG-amine (Creative PEGWorks) as previously reported^19^. The NanoBrook Omni (Brookhaven Instruments) was used to conduct dynamic light scattering (DLS) experiments to determine particle size distribution and surface charge. We confirmed the formation of a dense PEG coating on PS-NP based on its measured zeta potential of 0.04 ± 0.71 mV.

### Neuraminidase assay

The neuraminidase activity was tested using the NA-Fluor^TM^ Neuraminidase Assay Kit from Life Technologies and following manufacturer instructions. Fluorescence intensity was measured using a Spark Multimode Plate Reader (Tecan). A standard curve for 4-Methylumbelliferone (4-MU(SS); Sigma-Aldrich) was then generated and the fluorescence signal of 18,000 relative fluorescent units (RFU) corresponding to 20 µM 4-MU(SS) was chosen to normalize NA activity. The neuraminidase assay kit was also used to perform a neuraminidase inhibition assay using the neuraminidase inhibitor zanamivir (Cayman Chemical). IAV neuraminidase inhibition was tested using a 1:16 dilution of IAV and varying the concentration of zanamivir from 0.01 nM to 10 µM. In accordance with manufacturer instructions, IAV was incubated with zanamivir for 30 min before the NA-Fluor^TM^ substrate was added. The reaction was incubated for 1 h at 37 °C before fluorescence intensity was measured.

### Human mucus collection

Human mucus was collected under an IRB-approved protocol at the University of Maryland Medical Center (UMMC; Protocol #: HP-00080047). Samples were collected by the endotracheal tube (ET) method, as previously described^19^. ET were collected from 10 donors after intubation as a part of general anesthesia at UMMC. The data presented here are from 6 male and 3 female subjects with mean age of 66 ± 12 years (note: demographic data not available for 1 patient). All adults undergoing non-cardiothoracic surgery and under general anesthesia were eligible for the study. Given we were collecting a surgical waste product (endotracheal tubes), recruitment of participants was not required as the study was exempt from informed consent. In order to collect mucus from ET, the last approximately 10 cm of the tubes were cut, including the balloon cuff, and placed in a 50 mL centrifuge tube. The ET tube was suspended in the tube with a syringe needle and was then spun at 220 g for 30 s, yielding 100–300 μL of mucus. Mucus with visible blood contamination was not included in the analysis. Samples were stored at −80 °C immediately after collection and thawed (up to 3x) prior to use for experiments. Notably, control experiments showed the microrheology of an ET mucus sample was minimally impacted by freeze-thaw as compared to an unfrozen sample kept at 4 °C (Supplementary Fig. 9).

### Synthetic mucus hydrogel preparation

Using a previously established method^26^, synthetic mucus hydrogels were prepared using 2% bovine submaxillary mucin (BSM; Sigma-Aldrich) and varying percentages of a 10 kDa thiolated 4-arm polyethylene glycol (PEG-4SH; Laysan Bio Inc.) used as a thiosulfate crosslinker. The mucin and PEG-4SH crosslinker were combined in a physiological buffer (154 mM NaCl, 3 mM CaCl_2_, and 15 mM NaH_2_PO_4_ at pH 7.4) and mixed for 2 hours. The cross-linking solution was prepared separately in buffer directly before mixing with mucin solution. To initiate gelation, equal volume aliquots of each solution were mixed and equilibrated for 21 hours at room temperature. After synthetic mucus gels were fully formed, DiI-labeled IAV and/or nanoparticles were added prior to fluorescence video microscopy experiments. There were *n* = 3 gels tested for each cross-linking percentage, with a total of *n* ≥ 100 particles per condition.

### Fluorescence video microscopy

Samples were prepared for imaging by placing a vacuum grease coated O-ring on microscope cover glass. The sample was then applied to the center of the well and sealed with a coverslip. Samples were allowed to stabilize for 30 minutes at room temperature prior to imaging. Slides were imaged using Zeiss LSM 800 inverted microscope with a 63x water-immersion objective. For each sample, 10 s videos were recorded at 33.3 frames per second. For all experiments, 1 µL of DiI-labeled IAV (3 × 10^9^ PFU/mL) and 1 µL of PEG-coated nanoparticles were added to 20 µL thawed human mucus in the center of the slide well and stirred with a pipette tip prior to imaging. After equilibration and initial imaging, samples were incubated for 15 minutes at 37 °C and then imaged. For IAV with neuraminidase inhibitor (NAI) in human mucus, 1 µL of IAV labeled with DiI was mixed with 1 µL of NAI zanamivir (Cayman Chemical) and allowed to equilibrate for 10 minutes. The IAV and NAI mixture was then combined with 100 nm PS-NP and added to 20 µL of human mucus. The final concentration of NAI in the mucus sample with the IAV was 10 µM. For freeze-thaw testing, IAV was added to 20 µL of different aliquots of the same human mucus sample after 0, 1, 2, and 3 freeze-thaw cycles. For mucus treated with dithiothreitol (DTT; VWR), 1 µL of 115 mM DTT was added directly to the mucus sample, yielding a final concentration of 5 mM. On average, 137 IAV particles and 246 PS-NP were tracked per sample tested. In rare cases, a minimum of 10 IAV particles were tracked.

### Multiple particle tracking (MPT) analysis

Acquired fluorescence microscopy videos were processed using a previously developed MATLAB (The MathWorks, Natick, MA) based analysis code to isolate and track imaged particles^58–60^. For each video, the mean squared displacement (MSD) was calculated as $\left\langle {{\mbox{MSD}}}\left(\tau \right)\right\rangle =\left\langle \left({x}^{2}+{y}^{2}\right)\right\rangle$, for each particle. The MSD values of the particles were then used to determine the average *α* value for each sample, calculated as $\alpha ={{\mbox{(}}}\triangle {\log }{{\mbox{MSD}}}\left(\tau \right){{\mbox{)}}}/{{\mbox{(}}}\triangle {\log }\left(\tau \right){{\mbox{)}}}$. All experimental data were verified to have an average *α* value with 0 < *α* ≤ 1, indicative of sub-diffusive particle motion. Due to the nature of MPT, PS-NP and IAV were tracked for a maximum of 10 s due to their motion out of the focal plane. To minimize the dynamic and static error in our measurements^58–60^, a lag time of 1 s was used as a representative value for comparison between conditions. The MSD values for PS-NP were then used to calculate the microrheological properties of the samples tested using the generalized Stokes-Einstein relation^61^, defined as *G*(*s*) = 2*k*_B_*T/*(π*as*〈Δ*r*^2^(*s*)〉) gives the viscoelastic spectrum where *k*_*B*_*T* is the thermal energy, *a* is the radius, and *s* is the complex Laplace frequency^19^. The complex modulus is calculated by ${G}{\ast }\left(\omega \right)={G}^{\prime}\left(\omega \right)+{G}^{\prime\prime}{{\mbox{(}}}i\omega {{\mbox{)}}}$ where *iω* is used in place of *s*, *i* is a complex number, and *ω* is the frequency^19^. From this equation, the pore size (*ξ*) can be estimated as $\xi ={\left({k}_{B}T/G^{\prime}\right)}^{1{{{/}}}3}$ and the complex microviscosity (η*) can be calculated as η* = (G*(*ω*))/(*ω*)^19^. The effective diffusivity (*D*) was calculated for each individual particle using the MSD values as MSD = 4*D*τ^6^. The average effective diffusivity for each sample was then used to calculate the estimated diffusion time (*t*), using the equation $t\,={L}^{2}{{\mbox{/(}}}2D{{\mbox{)}}}$, where *L* is the thickness of the mucus layer^62^. For the estimated diffusion time, a physiological thickness of 7 µm was used for the calculations.

### Dissociation constant (K_3D_) for IAV binding to mucus

In order to quantify an effective binding constant for IAV within a mucus gel network, we have developed an analytical procedure that accounts for the confinement of IAV particles due to physical constraints (i.e., pore size limited, sub-diffusive motion) and adhesive interactions (i.e., HA-sialic acid binding). First, the trajectories from MPT analysis are recentered, based on the average *x-* and *y*-position, and the radial distance from the center of the trajectory (*r*) is calculated (in µm) for each frame. The *r* values for each frame are used to measure the confinement that a given particle experiences using the expression,

1 $$\sigma =\frac{2}{n}\mathop{\sum }\limits_{i=1}^{n}\left|{r}_{i}\right|$$

where *n* is the number of frames the trajectory is tracked over, and *σ* is the average diameter of the trajectory. A histogram is then constructed based on measured *r* for individual IAV and PS-NP particles. To determine an effective energy of confinement based on sampled *r*, histograms are used to calculate an energy of confinement (*U*_3D_) using a Boltzmann distribution as,

2 $${U}_{3{{{{{\rm{D}}}}}}}/{k}_{{{{{{\rm{B}}}}}}}T=-\,{{{{\mathrm{ln}}}}}[n(r)/{n}_{{{\max }}}]$$

where *n*(*r*) is the number of counts for a given *r* value, and *n*_max_ is the largest count value representing the most probable *r* position at the local energy minimum^63^. We consider the overall energy of confinement for IAV within mucus gels (*U*_3D,IAV_ = *U*_s_ *+ U*_B,IAV_) to be a result of the combined effects of steric obstruction (*U*_s_) and adhesive interactions due to binding (*U*_B,IAV_). Given the limited adhesive interactions expected for PS-NP, the overall confinement energies for PS-NP (*U*_3D,NP_) are averaged to account for confinement due to steric interactions to determine *U*_s_ as *U*_s_ = 〈*U*_3D,NP_〉. Importantly, 〈*U*_3D,NP_〉 for PS-NP within the same regions of interest as IAV are used to account for these effects as they will be depend on the local microenvironment within highly heterogenous mucus gels. The potential energy of IAV-mucus binding is then determined for individual IAV particles as *U*_B,IAV_ = *U*_3D,IAV_ − 〈*U*_3D,NP_〉.

Measured energy profiles for IAV-mucus binding (*U*_B,IAV_) for individual virions are then analyzed to estimate an effective dissociation constant using a generalized analytical expression for harmonic well potentials^25,64^. The spring constant (*k*_s_) is calculated by fitting measured *U*_B,IAV_ using the expression,

3 $${U}_{{{{{{\rm{B}}}}}},{{{{{\rm{IAV}}}}}}}/{k}_{{{{{{\rm{B}}}}}}}T=0.5({k}_{{{{{{\rm{s}}}}}}}/{k}_{{{{{{\rm{B}}}}}}}T){r}^{2},$$

where *k*_B_*T* is thermal energy in pN•nm and *k*_s_ is in units of pN/nm^63^. An effective dissociation constant for IAV binding to the mucus gel network, *K*_3D_, is then calculated as,

4 $${K}_{{{{{{\rm{3D}}}}}}}={(2{{{{{\rm{\pi }}}}}}{k}_{{{{{{\rm{B}}}}}}}T/{k}_{{{{{{\rm{s}}}}}}})}^{-3/2}\exp (-{U}_{{{{{{\rm{M}}}}}}}/{k}_{{{{{{\rm{B}}}}}}}T),$$

where *U*_M_ is the depth of energy well for measured *U*_B,IAV_ (in pN•µm)^25^. Given these estimates are determined for viral particles with multiple receptors diffusing in a 3D biological gel decorated with ligands, these effective dissociation constants will likely differ from those determined using traditional single molecule-based assays. Thus, we believe it is important to distinguish the estimated dissociation constant here from traditional measurements and we have denoted the dissociation constant with the symbol “*K*_3D_” to reflect the nature of our measurements. In our previous work, this analytical expression was computationally validated and produces accurate dissociation constants for harmonic well potentials of varying depth and range^25^. These calculations were not performed on *U*_B,IAV_ energy profiles with *U*_M_ values less than 1 *kT* and negligible binding.

### Statistics and reproducibility

The number of patient samples used in our study were based on the number available for collection during the study. Studies in synthetic hydrogels were performed in triplicate. For each sample and treatment, PS-NP and IAV particles from at least *n* = 3 microscopy videos were tracked and pooled for analysis. Differences in measured parameters were calculated using either non-parametric two-tailed Mann-Whitney test or non-parametric Kruskal-Wallis test and Dunn’s test for multiple comparisons. The resulting *p*-values were considered significant if *p* < 0.05. Data were statistically analyzed using GraphPad Prism 9 (GraphPad Software, San Diego, CA).

### Reporting Summary

Further information on research design is available in the Nature Research Reporting Summary linked to this article.

## Supplementary information

Supplementary Information

Description of Additional Supplementary Files

Supplementary Data 1

Reporting Summary

## Data Availability

Source data for generation of main figures is available in Supplementary Data 1. Additional data can be made available upon reasonable request.

## Code availability

The particle tracking code used in the current work is available at http://site.physics.georgetown.edu/matlab/. The code used to determine microrheological properties was adapted from previously published code, available at http://www.physics.emory.edu/faculty/weeks/idl/rheo. Custom codes used in this study to estimate dissociation constant were run on MATLAB software (version R2020b) and are posted on Zenodo (10.5281/zenodo.4697578)^65^.
