# Supplementary material for: Influenza A virus diffusion through mucus gel networks
Source: Commun Biol. 2022 Mar 22;5:249. doi: 10.1038/s42003-022-03204-3 (PMC8941132; doi:10.1038/s42003-022-03204-3)
Supplement: Supplementary file 1 — Supplementary Information [file 42003_2022_3204_MOESM1_ESM.pdf]

Influenza A virus diffusion through mucus gel networks

Logan Kaler[1], Ethan Iverson[2], Shahed Bader[3], Daniel Song[3], Margaret A. Scull[2], Gregg A. Duncan[1,3]
[1]Biophysics Program, [2]Department of Cell Biology & Molecular Genetics, and [3]Fischell Department of Bioengineering, University of Maryland, College Park, MD 20742, USA

SUPPLEMENTAL INFORMATION

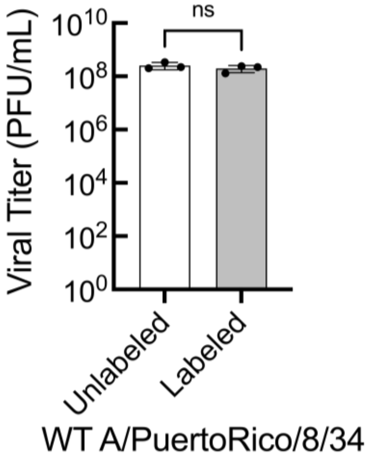

Figure S1. Infectivity of IAV with and without labeling with DiI. Plaque forming units (PFU) of WT A/PuertoRico/8/34 (H1N1) were measured before (white) and after (grey) labeling with lipophilic dye, 1,1'-dioctadecyl-3,3,3'3'-tetramethylindocarbocyanine perchlorate (DiI).

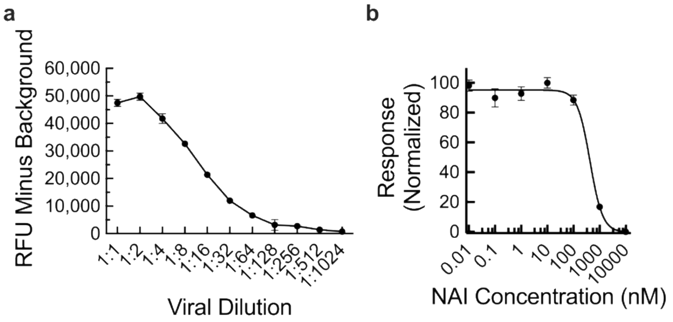

Figure S2. Neuraminidase activity and inhibition assay for unlabeled IAV. **a**) Neuraminidase activity at different viral dilutions of unlabeled IAV. **b**) Neuraminidase activity of unlabeled IAV (1:16 dilution) in varying concentrations of neuraminidase inhibitor Zanamivir.

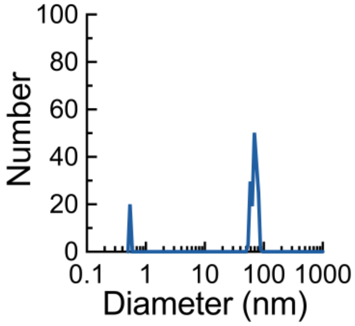

Figure S3. Dynamic light scattering for IAV with neuraminidase inhibitor Zanamivir. Size of particles were measured after IAV was mixed with the neuraminidase inhibitor Zanamivir in the same concentrations used in the mucus samples.

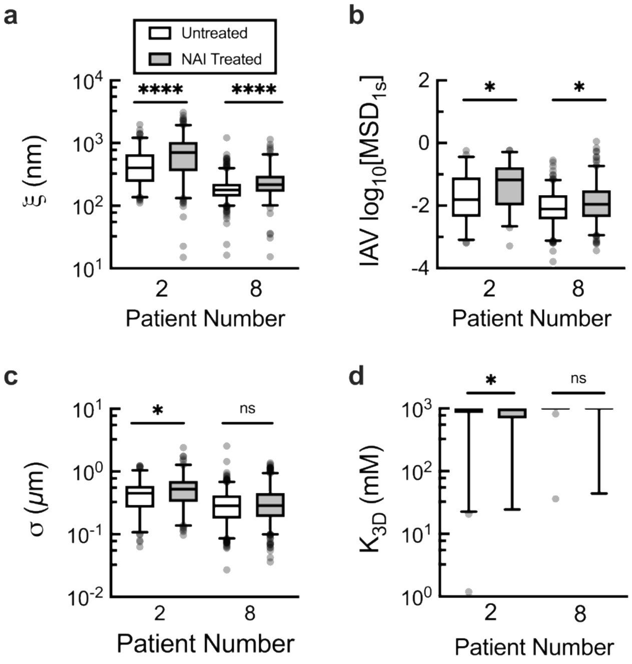

Figure S4. Neuraminidase inhibition and IAV diffusion in samples with altered microstructure. **a**) Calculated pore size (ξ) in untreated (white) and NA inhibitor (NAI) treated (zanamivir; 10 µM final concentration; grey) based on PS-NP diffusion in human mucus. **b**) Measured $\log_{10}MSD_{1s}$ for IAV diffusion in untreated and NAI treated human mucus. Box and whisker plots of **c**) average trajectory diameter (σ) and **d**) calculated dissociation constants with and without NAI treatment. Whiskers are drawn down to the 5th percentile, up to the 95th percentile, and outliers are plotted as points. Data set statistically analyzed with two-tailed Mann-Whitney test: ns = not significant; $p > 0.05$, *$p < 0.05$, **$p < 0.01$, ***$p < 0.001$, ****$p < 0.0001$. Patient numbers correspond with those in Figures 2 and 3.

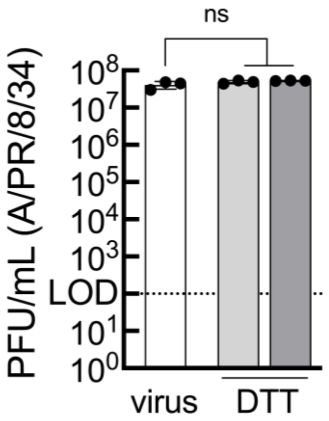

Figure S5. Effect of DTT on IAV infectivity. Plaque forming units (PFU) of A/PR/8/34 (H1N1) were measured before (white) and after treatment with 5 mM (light grey) and 10 mM (dark gray) dithiothreitol (DTT). Equivalent titers of virus were incubated at 37C in serum free DMEM with DTT at indicated concentrations for 30 minutes prior to plaque assay.

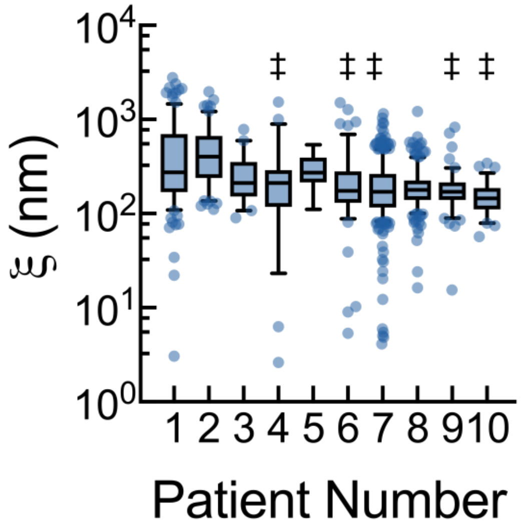

Figure S6. Measured pore size of human mucus. Box-and-whisker plots of pore size (ξ) at τ = 1 s for PS-NP (blue circles) in mucus samples collected from 10 individual patients. Double dagger (‡) indicates sample in which the $\log_{10}[MSD]_{1s}$ for IAV is greater than the $\log_{10}[MSD]_{1s}$ for PS-NP. Whiskers are drawn down to the 5th percentile, up to the 95th percentile, and outliers are plotted as points.

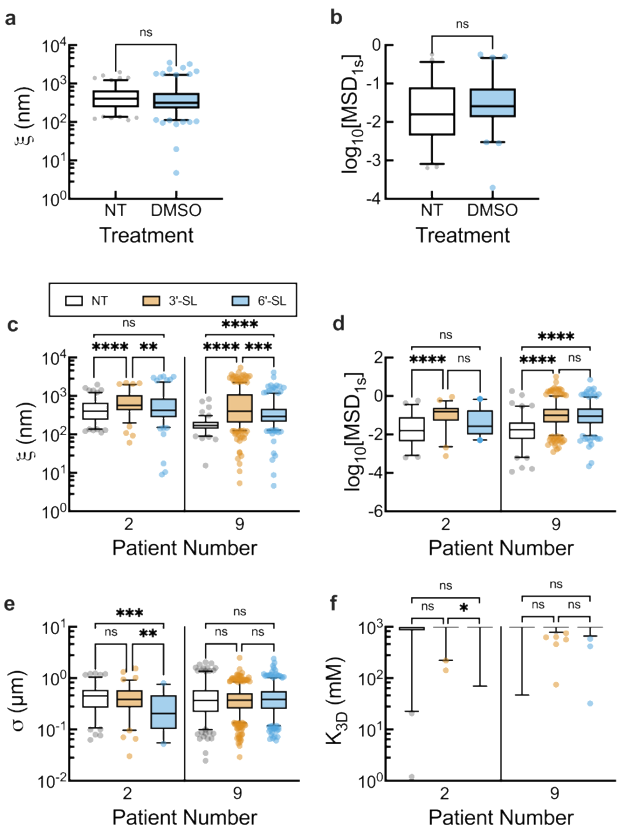

Figure S7. 100nm PS-NP and IAV in human mucus following treatment with sialyllactose. For each treatment, 1 µL of IAV was combined with 1 µL of pure DMSO, 400 mM 3'-sialyllactose (3'-SL; f.c. 200 mM), or 400 mM 6'-sialyllactose (6'-SL; f.c. 200 mM)). The IAV mixture equilibrated at room temperature for 10 minutes before it was introduced 20 µL of human mucus along with 1 µL of PS-NP. The distribution of the pore size (ξ) and $\log_{10}[MSD]_{1s}$ values for untreated IAV and IAV treated with DMSO are shown as box and whisker plots in (**a**) and (**b**), respectively. The distribution of the pore size (ξ) and $\log_{10}[MSD]_{1s}$ values for untreated IAV and IAV treated with 3'-SL or 6'-SL are shown as box and whisker plots in (**c**) and (**d**), respectively. Distribution of the average trajectory diameter (σ) IAV and dissociation constant (K_{3D}) are plotted in (**e**) and (**f**), respectively. Whiskers are drawn down to the 5th percentile, up to the 95th percentile, and outliers are plotted as points. Data in **a**-**b** statistically analyzed with two-tailed Mann-Whitney test: ns = not significant; $p > 0.05$, *$p < 0.05$, **$p < 0.01$, ***$p < 0.001$, ****$p < 0.0001$. Data in **c**-**f** statistically analyzed with Kruskal-Wallis test and Dunn's test for multiple comparisons: ns = not significant; $p > 0.05$, *$p < 0.05$, **$p < 0.01$, ***$p < 0.001$, ****$p < 0.0001$. Patient numbers correspond with those in Figures 2 and 3.

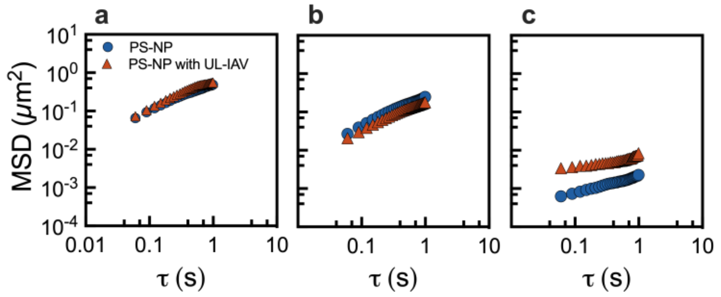

Figure S8. 100nm PS-NP and unlabeled IAV in human mucus samples. **a-c**) MSD of PS-NP (blue circles) and PS-NP in the presence of unlabeled IAV (red triangles) dispersed in three human mucus samples.

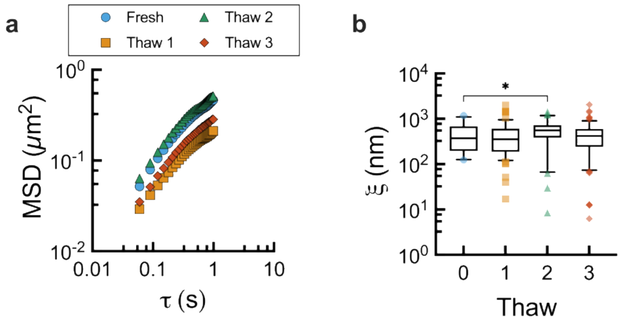

Figure S9. Human mucus sample with varying freeze-thaws. **a**) MSD of particles dispersed in a fresh (blue circles) human mucus sample and after 1, 2, and 3 freeze-thaw cycles (orange squares, green triangles, and red diamonds, respectively). **b**) Calculated pore size (ξ) for individual particles at $\tau = 1$ s. Whiskers are drawn down to the 5th percentile, up to the 95th percentile, and the outliers are plotted as points. Data set statistically analyzed with Kruskal-Wallis test and Dunn's test for multiple comparisons: *$p < 0.05$, **$p < 0.01$, ***$p < 0.001$, ****$p < 0.0001$.
