# Supplementary material for: Influenza A virus diffusion through mucus gel networks
Source: Commun Biol. 2022 Mar 22;5:249. doi: 10.1038/s42003-022-03204-3 (PMC8941132; doi:10.1038/s42003-022-03204-3)
Supplement: Supplementary file 2 — Description of Additional Supplementary Files [file 42003_2022_3204_MOESM2_ESM.pdf]

Description of Additional Supplementary Files

File name: Supplementary Data 1
Description: Source data used to generate all graphs presented in the main figures.
